# Sexual and Physical Victimization and Health Correlates Among Norwegian Adolescents

**DOI:** 10.1007/s10508-023-02604-8

**Published:** 2023-05-08

**Authors:** Lars Roar Frøyland, Willy Pedersen, Kari Stefansen, Tilmann von Soest

**Affiliations:** 1 NOVA – Norwegian Social Research, OsloMet – Oslo Metropolitan University, St. Olavs Plass, P. O. Box 4, 0130 Oslo, Norway; 2 Department of Sociology and Human Geography, University of Oslo, Oslo, Norway; 3 Department of Psychology, PROMENTA Research Center, University of Oslo, Oslo, Norway

**Keywords:** Sexual health, Mental health, Sexual victimization, Physical victimization

## Abstract

Large-scale epidemiological studies have documented that many children and adolescents are exposed to different forms of victimization experiences. However, such population-based studies have rarely examined how specific types of victimization are correlated with health indicators. Thus, we investigated sexual victimization, physical victimization by parents, and physical victimization by peers and their associations with sexual health, mental health, and substance use. We gathered data from a nationally representative sample of Norwegian 18–19-year-old students in their final year of senior high school (*N* = 2075; 59.1% girls). The analyses showed that 12.1% of the adolescents reported sexual victimization experiences. Physical victimization was more prevalent: 19.5% of the respondents had been exposed to victimization from parents and 18.9% from peers. Multivariate analyses revealed specific associations between sexual victimization and a range of sexual health indicators, such as early sexual intercourse debut, many sexual partners, engaging in sex without contraception while intoxicated, and participating in sexual acts for payment. Neither physical victimization from parents nor from peers were correlated with these variables. However, all three forms of victimization were associated with impaired mental health and potential substance use problems. We conclude that a variety of victimization experiences should be addressed in policies for prevention of adolescent mental health and substance use problems. In addition, a special emphasis is warranted regarding sexual victimization: Sexual health policies should address such potential experiences in addition to more traditional themes such as reproductive health and should also include low-threshold services for young victims of sexual victimization.

**Supplementary Information:**

The online version contains supplementary material available at 10.1007/s10508-023-02604-8.

## Introduction

Large-scale epidemiological studies have documented that many children and adolescents are exposed to different forms of victimization experiences. For example, the U.S. National Survey of Children’s Exposure to Violence (Finkelhor et al., 2015) identified past-year prevalence rates of 5% for sexual victimization, 16% for parental physical victimization, and 5% for peer physical victimization among children from birth to age 18. Similarly, the cross-national Violence Against Children Surveys (Chiang et al., 2016) reported lifetime prevalence rates of over 20% for sexual victimization and 50% for physical victimization in eight participating countries in Africa, the Caribbean, and Southeast Asia. However, such population-based studies have rarely examined the complex interplay of several types of victimization experiences and indicators of health. We address this issue by examining data from a population-based study among Norwegian adolescents to determine the associations between different forms of victimization and sexual health, mental health, and substance use.

In the current study, we particularly focused on the relationship between victimization and sexual health. Sexual health is an evolving concept that has been expanded from the original definition given by the World Health Organization in 1975 (Edwards & Coleman, 2004). From mainly focusing on sexual risk, it now includes concepts of mental health, responsibility, and sexual rights. In accordance with this development, early research on sexual health typically focused primarily on adverse outcomes, such as unplanned pregnancies and sexually transmitted diseases. However, in the context of critical scholarship on youth sexual health, Naezer (2018) and Shoveller and Johnson (2006) have argued for using a more comprehensive approach than the narrow focus on “sexual risk.” Thus, in this study, we explored a broad set of sexual health indicators.

In an effort to advance this more comprehensive research agenda, we collected data on three prevalent forms of victimization in adolescents’ lives: sexual victimization, physical victimization from parents, and physical victimization from peers. We examined the association between these three forms of victimization and a variety of sexual health measures and indicators of impaired mental health and substance use problems.

### Victimization and Health Correlates

Sexual victimization has gradually become recognized as an important element of adolescents’ lives (A. M. Young et al., 2009). Such experiences often happen in party settings involving the excessive consumption of alcohol or other drugs (Stefansen et al., 2021; Tutenges et al., 2020) and may trigger stigmatizing reactions from others, leading victims to self-blame and feel ashamed (Kennedy & Prock, 2018). Sexual victimization, which has been widely researched over the past few decades, seems to be more prevalent among adolescents than adults (A. M. Young et al., 2009). Moreover, research has consistently documented substantially higher victimization rates among girls than among boys (A. M. Young et al., 2009). For example, a population-based study of adolescents from the USA showed that twice as many girls as boys reported having experienced forced sexual intercourse (Howard et al., 2007). Even greater gender differences were revealed among U.S. high school students, with 12% of girls and 3% of boys reporting sexual victimization experiences (A. M. Young et al., 2009). A similar gender difference was reported in a recent study of Norwegian high school students aged 18–19 years, where 14% of girls and 3% of boys had experienced serious and forced sexual victimization or rape (Stefansen et al., 2019). Most sexual assaults are committed by someone the victim already knows (Rennison, 2002), among others in the context of dating or an established relationship (Taylor & Mumford, 2016).

Systematic reviews and meta-analyses (Abajobir et al., 2017; Senn et al., 2008) have identified consistent associations between childhood and adolescent sexual victimization and certain sexual health indicators, such as having more sexual partners, early intercourse debut, and engaging in sex for payment. Such indicators may be interpreted as markers of sexual risk behaviors, but studies have also highlighted that early and frequent sexual activity may be related to a positive and healthy adult sexuality (Harden et al., 2008). An Australian study based on a nationally representative sample also showed how childhood sexual abuse was related to a significant increase in the frequency of sexual dysfunction symptoms in adulthood (Najman et al., 2005).

Sexual victimization in childhood and adolescence has also been linked to a broad spectrum of indicators of mental health problems, such as anxiety, depression, and post-traumatic stress disorder (PTSD), as documented by meta-analyses (Hailes et al., 2019) and reviews particularly targeting community samples (Lindert et al., 2014). Analyses of nationally representative samples from Australia (Najman et al., 2007b) and Israel (Mansbach-Kleinfeld et al., 2015) have provided similar findings, even though studies also find complete mental health in adult life among a sizeable proportion of forced child sexual abuse victims (Fuller-Thomson et al., 2020). Among adolescents, problematic alcohol use and increased use of other substances are often associated with sexual victimization (Champion et al., 2004). However, a common challenge in many studies is that voluntary sexual experiences that represent statutory violations are often included together with forced or coerced sexual victimization experiences when measuring sexual abuse.

Also common in the lives of adolescents are other forms of victimization, such as physical victimization from parents (Chan et al., 2021) and from peers (Reijntjes et al., 2010). Much research has assessed whether adolescents have experienced a particular form (i.e., physical or sexual) of victimization. However, if we are to understand how victimization, sexual health, and other potentially negative health indicators are linked, adolescents’ exposure to different forms of victimization must be considered simultaneously (Hamby et al., 2018). In addition to research on health-related outcomes of sexual victimization, a range of studies has mapped adverse health outcomes of other forms of victimization, such as child maltreatment and bullying (see, e.g., Gilbert et al., 2009; Moore et al., 2017). Studies within this tradition have shown that peer victimization is linked to depression and anxiety (Reijntjes et al., 2010), substance abuse (Brady & Donenberg, 2006), and “off-time” (e.g., early/precocious) life-course transitions, such as dropping out of high school and teenage pregnancies (Hagan & Foster, 2001). Research has also shown that maltreatment and physical victimization by parents are related both to mental health problems (Chan et al., 2021) and sexual health indicators such as early sexual onset, substance use during sex, higher number of sexual partners, and adolescent pregnancies (Rodgers & McGuire, 2012).

Two Swedish studies explored sexual health related to various forms of victimization. A cross-sectional study of users of Swedish youth clinics (Hammarström et al., 2022) showed that those who reported a combination of physical and sexual victimization had higher rates of early sexual intercourse, sexual risk-taking, and alcohol and substance use compared to their non-victimized peers. Similarly, evidence from a study among Swedish high school students that covered a variety of sexual behaviors among victims of physical, sexual, and emotional victimization (Blom et al., 2016) demonstrated similar associations between experiences of different forms of victimization and a range of sexual health indicators, such as unintended pregnancies, unprotected sex, early intercourse debut, and a high number of sex partners. Neither of the studies investigated whether correlates for different forms of victimization varied for health indicators in other domains than sexual health. This is what we aimed to examine in the present study.

Both victimization and the selected health indicators in this study are related to a wide range of sociodemographic characteristics. Studies have highlighted that socioeconomic resources, migration background, and parental divorce are associated with both the occurrence of victimization and physical and mental health (Gilbert et al., 2009; Moore et al., 2017). Thus, these characteristics were included as potential confounders in this study. Additionally, individual differences in adolescent sexual behavior may be attributed to genetic differences (Harden, 2014). The current study did not contain information on genes, but perceived pubertal timing was included as a proxy measure of biological influences on sexual behavior.

### The Norwegian Context

Norway is one of the Nordic welfare states, where health policy development is often characterized by an emphasis on equity and social security through universal strategies (Raphael, 2014). These characteristics are also evident in policies designed to ensure better sexual health (Ministry of Health & Care Services, 2016). Over the past decade, emphasis on the importance of sexuality for a good life has increased. For instance, the latest governmental strategy on youth health (2016–2021) included a section on sexual health. The section focuses not only on teenage pregnancies, contraceptives, sexually transmitted diseases, and abortion rates but also on broader dimensions of sexual identity and integrity (Ministry of Health & Care Services, 2016). In line with this thinking, adolescents in Norway have access to specialized youth sexual clinics, which are available at least in large cities and offer medical services and counseling at a low cost. Contraceptives are subsidized for those under the age of 22 years, and youths do not need parental permission to obtain them.

Norway is considered a liberal country in terms of sexual norms, although some subpopulations take a more restrictive outlook on sexuality. The legal age of consent for sex is 16 years, and estimates on the average sexual debut age in Norway show that youths typically engage in sexual activity prior to cohabitation and marriage (Hansen et al., 2020).

### The Present Study

For the present study, we gathered data from a nationally representative sample of students in their final year of senior high school in Norway. We studied associations between adolescents’ experiences with sexual victimization (defined as forced/coerced sexual encounters), physical victimization by parents, and physical victimization by peers and a range of indicators related to sexual health, mental health, and substance use. We asked: Are such associations of a more *general* character, implying that all forms of victimization are related to all health indicators similarly? Or do we observe more *specific* associations, implying that certain forms of victimization are more or less closely associated with specific indicators? In addition, we investigated whether including a wide range of potential confounders would reduce the magnitude of the observed associations.

## Method

### Participants and Procedure

For the present study, questionnaire data from the *UngVold* 2015 study of final-year senior high school students in Norway were examined. The study was designed to map experiences with victimization and violence among Norwegian adolescents. The initial size of the study sample was, thus, scaled to facilitate valid assessments of potentially low-prevalent phenomena. The survey was conducted in 2015, and schools were drawn from a pool of all senior high schools in Norway to obtain a nationally representative sample. The school sample was stratified according to geographical region to ensure participation from all parts of the country. Each school’s sampling probability was proportional to the number of enrolled students. We selected 61 schools, of which 36 agreed to participate. Five additional schools were strategically recruited to ensure a nationally representative school sample despite of declining schools. All students in their final year of senior high school at the selected schools were asked to participate in the study by their teachers and were provided with information about the study both orally and in writing. Informed consent was obtained prior to study participation. The response rate for the survey was 66%. The response rate did not vary according to gender or geographical region of the country, so no sampling weights were used. The participating students primarily attended study programs preparing them for enrollment in higher education (78% in general studies and 17% in vocational studies normally followed by higher education). Students provided their responses to the online questionnaire in a classroom during two school hours in the presence of a teacher. To prevent the possibility that answers to highly sensitive questions could be visible to other students in the class, the schools were instructed to administer the surveys as they would administer an examination.

The analyses in this paper were restricted to 18–19-year-old students, indicating that they were following an expected trajectory through high school. Due to a methodological experiment on how to measure rape (Stefansen et al., 2019), only half of the respondents were given the opportunity to respond to the questionnaire that included all the items on victimization used in this study. The final analytic sample consisted of 2075 students (59.1% girls).

### Measures

#### Sexual Victimization

Sexual victimization was measured using an instrument consisting of seven items separating victimization experiences before and after the age of 13, respectively, with three response options: *no, never* (0), *yes, once* (1), and *yes, more than once* (2). The severity of the experiences ranged from being pressured into sexual activities against the respondent’s will to acts constituting rape in the penal code. The items were combined into a sum score with range 0–28. The instrument on sexual victimization was also transformed into a binary variable separating those reporting no sexual victimization from those reporting at least one instance of sexual victimization.

#### Physical Victimization

Physical victimization from both parents and peers was assessed. A 14-item instrument was used to measure parental physical victimization, modeled after the Parent–Child Conflict Tactics Scale (Straus et al., 1998). Seven items assessed the frequency of different acts of violence by the respondents’ mothers and fathers, respectively. Response options ranged on a six-point Likert scale as follows: *never* (0), *once* (1), *a few times* (2), *monthly* (3), *weekly* (4), and *daily* (5). The items were combined into a sum score with the range 0‒70. Physical victimization from peers was measured using a six-item instrument assessing respondents’ experiences with three different acts of physical violence either before or after the age of 13, where the perpetrator was “someone your own age,” with the same response options as the instrument on physical victimization from parents. The items were combined into a sum score ranging from 0 to 30. Similar to the instrument on sexual victimization, peer and parental physical victimization were also transformed into binary variables separating those reporting no victimization from at least one victimization experience. See Supplementary Table 1 for an overview of all the items used to measure victimization.

#### Sexual Health

Early sexual debut was measured by self-reports about age of sexual intercourse debut, in which those with first sexual intercourse before the age of 15 were contrasted with other respondents. We defined a high number of sexual partners as having had four or more sexual partners during the previous 12 months. Moreover, a dichotomous item measured whether the respondents had had sex without contraception while intoxicated during the previous 12 months. Finally, a single item separated respondents that had ever participated in sexual acts for payment from the remainder of the respondents.

#### Mental Health and Substance Use

Emotional distress was measured by the average score of a 25-item version (*α* = 0.94) of the Hopkins Symptom Checklist (HSCL-25; Parloff et al., 1954). The respondents answered questions on depressive symptoms (e.g., “feeling hopeless about the future”) and anxiety (e.g., “suddenly scared for no reason”) in the previous week on a scale ranging from 1 (not at all distressed) to 4 (very much distressed). Posttraumatic stress (PTSD) was measured by a seven-item screening instrument (*α* = 0.80) inspired by the diagnostic criteria for PTSD in the DSM-IV (Breslau et al., 1999). The respondents answered “yes” or “no” to ever having had an experience that resulted in affirmative answers to diagnostic PTSD symptoms during the previous month (e.g., “Lost interest in activities that you liked to do or that used to be important to you?” and “Felt that planning for the future is of no use?”). The final instrument counted the number of positive answers (range 0–7).

To measure problematic alcohol use, a six-item instrument (*α* = 0.73) was used, modeled after the Rutgers Alcohol Problems Index (RAPI; White & Labouvie, 1989). The instrument covered instances of different alcohol-related problems of the participants during the previous 12 months, and the response options were as follows: *none* (1), *once* (2), *2‒4 times* (3), *5‒10 times* (4), and *more than 10 times* (5). Examples of items are “Argued with or insulted someone” and “Vomited because you had drunk too much.” The instrument was coded as the mean score of the included items. Moreover, we assessed the use of cannabis, other narcotics, or intentional intoxication on prescription drugs in the previous 12 months. The instrument was dichotomized into “use” versus “no use.”

#### Confounders

Gender was assessed by self-report. To measure the socioeconomic status of the participants’ families, we assessed whether at least one parent of the respondent had a university education and whether at least one parent worked full time. Additionally, the respondents reported their subjective view of their family’s economic status during the prior 2 years on a five-point Likert scale. The respondents were also asked whether their parents were divorced and whether they had two foreign-born parents. Parental alcohol use was measured using the highest scores on a five-point Likert scale to indicate the number of times respondents had seen their mother or father, respectively, intoxicated. Finally, a single item assessed pubertal timing using a seven-point Likert scale, which the respondents used to indicate whether they perceived puberty to have commenced earlier or later for them than it had for others of the same gender and age group.

### Statistical Analyses

To obtain comparable regression coefficients across different predictor variables, we recoded the instruments on sexual and physical victimization such that all three variables ranged from 0 (no form of victimization experience) to 10 (maximum achievable score on the scale). Then, linear and logistic regression analyses were mapped onto the continuous and binary indicators, respectively. The analyses consisted of the following three steps: (1) we estimated bivariate associations between victimization and all health correlates separately for each type of victimization, (2) we conducted multivariate regression analyses in which we additionally controlled for potential confounders, and (3) we included all three victimization instruments and confounders together as predictors in the regression analyses. The estimates from the linear regression analyses are presented as unstandardized regression coefficients (*b*). The estimates from the logistic regression analyses are presented as odds ratios (*OR*). To account for potential gender differences in the associations between victimization and the included health indicators, initial analyses contained interaction terms between gender and different forms of victimization. The analyses returned few significant interactions (*p* > .05), so we chose to present the relevant interaction effects in the text rather than perform all analyses separately for girls and boys.

The analyses were conducted in R 4.1 (R Core Team, 2020). Missing data were handled using multiple imputation on 20 imputed data sets with the mice package in R (van Buuren & Groothuis-Oudshoorn, 2011), which is in accordance with state-of-the-art recommendations for handling this issue (Enders, 2022). The frequency of missing data was low for all included instruments (< 6%). The data used in the study, as well as the fully reproducible code of all analyses, are available at https://osf.io/d8fcq/.

## Results

The results showed that 12.1% of the sample reported having experienced sexual victimization, whereas 19.5% and 18.9% reported physical victimization by their parents and peers, respectively. Sexual victimization was much more common among girls than among boys, with rates of victimization being 17.8% and 3.6%, respectively (*p* < .001). Girls and boys reported similar levels of physical victimization from parents (girls = 19.6%, boys = 19.5%; *p* = .960) while 27.6% of all boys reported at least some peer physical victimization as opposed to 13.0% of girls (*p* < .001).

The scores for the three victimization instruments in Table 1 indicate that frequent victimization was relatively rare, with mean scores of 0.15 for sexual victimization, 0.11 for physical victimization from parents, and 0.30 for peer victimization (measured on a scale from 0 to 10). The correlations between the three instruments of victimization were small to medium, according to the guidelines suggested by Cohen (1988). The strongest correlations between victimization and the included health indicators were observed for instruments measuring aspects of mental health problems, while physical victimization by parents and peers was uncorrelated with several measures of sexual health. The correlations were considered small according to Cohen’s guidelines (1988).

**Table 1:** Descriptive statistics and correlations for variables under study

	*M*	SD	Median	Correlations									
				1	2	3	4	5	6	7	8	9	10
*Victimization experiences*													
1. Sexual victimization (0–10)	0.15	0.67	0.00										
2. Parental physical victimization (0–10)	0.11	0.40	0.00	.33									
3. Peer physical victimization (0–10)	0.30	1.01	0.00	.15	.27								
*Sexual health*													
4. Sexual debut before age 15, %	13.8			.16	.04	.06							
5. 4 + sexual partners in the previous 12 months, %	8.6			.07	.01	.04	.12						
6. Sex without contraception while intoxicated, %	31.2			.05	.04	.04	.09	.32					
7. Sex for payment, %	4.3			.09	.06	.02	.03	.07	.05				
*Mental health*													
8. Emotional distress (1–4)	1.66	0.55	1.52	.22	.17	.19	.12	.10	.08	.03			
9. Posttraumatic stress (0–7)	0.80	1.51	0.00	.22	.20	.20	.11	.09	.06	.04	.55		
*Substance use*													
10. Alcohol-related problems (1–5)	1.41	0.44	1.29	.11	.11	.15	.12	.25	.44	.08	.16	.15	
11. Other substance use, %	13.5			.07	.10	.09	.05	.19	.22	.02	.11	.11	.32

Correlations of *r* =|.06| and above are significantly different from 0 at *p* < .05

Tables 2 and 3 present linear and logistic regression analyses for all included health measures. The first rows of the tables show bivariate regression analyses between the three victimization instruments and the selected health indicators. The interpretation of the coefficients is the amount of change in the respective variable with a 10% increase in the victimization score. Sexual victimization was significantly related to all included indicators of sexual health and was found to be related to a higher prevalence of early sexual intercourse debut, a high number of sexual partners, having sex without contraception while intoxicated, and having received payment for sexual acts. Moreover, sexual victimization was adversely related to emotional distress, posttraumatic stress, problematic alcohol use, and other substance use. Both parental and peer physical victimization were also significantly associated with all mental health and substance use variables, while there were few associations with the sexual health indicators. In the second set of analyses presented in Tables 2 and 3, estimates were controlled for a range of relevant confounders. Overall, such control resulted in small changes in the regression estimates, and all but one of the significant associations from the bivariate analyses remained significant (the association between sexual victimization and having had sex without contraception while intoxicated). Finally, in addition to all confounders, all three forms of victimization were included simultaneously as independent variables in the multivariate analyses. In these models, sexual victimization was still associated with all health correlates except having had sex without contraception while intoxicated. Parental and peer physical victimization were also still associated with all indicators of impaired mental health and substance use problems, but they were not associated with any sexual health indicators.

**Table 2:** Regression analyses with sexual health indicators as dependent variables and victimization and confounders as independent variables

	Sexual debut before age 15	4 + sexual partners in the previous 12 months	Sex without contraception while intoxicated	Sexual acts for payment
	*OR* (95% CI)	*OR* (95% CI)	*OR* (95% CI)	*OR* (95% CI)
*Separate analyses for each type of victimization*				
Sexual victimization	1.59 (1.29; 1.97)***	1.27 (1.09; 1.48)**	1.17 (1.01; 1.35)*	1.34 (1.10; 1.62)**
Parental physical victimization	1.27 (0.90; 1.80)	1.10 (0.80; 1.52)	1.20 (0.92; 1.57)	1.40 (0.96; 2.05)
Peer physical victimization	1.14 (1.02; 1.29)*	1.11 (0.98; 1.25)	1.09 (0.99; 1.19)	1.09 (0.92; 1.29)
*Separate analyses for each type of victimization with control for confounders*				
Sexual victimization	1.50 (1.21; 1.87)***	1.23 (1.04; 1.44)*	1.15 (0.98; 1.33)	1.33 (1.08; 1.63)**
Parental physical victimization	1.14 (0.75; 1.73)	0.98 (0.67; 1.42)	1.06 (0.97; 1.17)	1.26 (0.85; 1.88)
Peer physical victimization	1.15 (1.01; 1.31)*	1.09 (0.96; 1.24)	1.11 (0.84; 1.46)	1.07 (0.90; 1.27)
*Analyses with all types of victimization included simultaneously, with control for confounders*				
Sexual victimization	1.51 (1.20; 1.89)***	1.26 (1.06; 1.50)**	1.13 (0.97; 1.32)	1.29 (1.04; 1.60)*
Parental physical victimization	0.83 (0.49; 1.41)	0.79 (0.54; 1.17)	1.00 (0.73; 1.36)	1.13 (0.71; 1.80)
Peer physical victimization	1.12 (0.96; 1.31)	1.09 (0.95; 1.24)	1.05 (0.95; 1.16)	1.02 (0.83; 1.26)

***p < .001; **p < .01; *p < .05. 95% CI = 95% confidence interval of *OR*

**Table 3:** Regression analyses with mental health and substance use indicators as dependent variables and victimization and confounders as independent variables

	Emotional distress	Posttraumatic stress	Alcohol-related problems	Other substance use
	*b* (95% CI)	*b* (95% CI)	*b* (95% CI)	*OR* (95% CI)
*Separate analyses for each type of victimization*				
Sexual victimization	0.19 (0.14; 0.23)***	0.49 (0.38; 0.60)***	0.07 (0.04; 0.11)***	1.73 (1.19; 2.51)**
Parental physical victimization	0.23 (0.15; 0.31)***	0.30 (0.23; 0.36)***	0.07 (0.05; 0.09)***	1.21 (1.10; 1.34)***
Peer physical victimization	0.10 (0.08; 0.13)***	0.75 (0.55; 0.94)***	0.12 (0.05; 0.19)***	1.24 (1.06; 1.46)**
*Separate analyses for each type of victimization with control for confounders*				
Sexual victimization	0.14 (0.10; 0.18)***	0.42 (0.31; 0.53)***	0.07 (0.04; 0.11)***	1.54 (1.03; 2.28)*
Parental physical victimization	0.18 (0.10; 0.26)***	0.64 (0.44; 0.83)***	0.09 (0.02; 0.16)**	1.18 (1.06; 1.31)**
Peer physical victimization	0.11 (0.09; 0.13)***	0.29 (0.22; 0.36)***	0.06 (0.04; 0.07)***	1.33 (1.12; 1.59)**
*Analyses with all types of victimization included simultaneously, with control for confounders*				
Sexual victimization	0.11 (0.07; 0.14)***	0.31 (0.20; 0.42)***	0.06 (0.03; 0.09)***	1.26 (1.05; 1.51)*
Parental physical victimization	0.07 (−0.01; 0.15)	0.33 (0.12; 0.53)**	0.03 (−0.04; 0.10)	1.27 (0.82; 1.95)
Peer physical victimization	0.09 (0.07; 0.11)***	0.23 (0.16; 0.30)***	0.05 (0.03; 0.07)***	1.13 (1.01; 1.26)*

***p < .001; **p < .01; *p < .05. *OR* = odds ratio. 95% CI = 95% confidence interval

To assess whether the associations differed between girls and boys, a final set of analyses included interaction terms for gender and different forms of victimization, as well as all confounders. For PTSD, the analyses returned a significant interaction term for gender and sexual victimization (*b* = 0.51; *p* = .004) and gender and parental physical victimization (*b* = 1.17; *p* < .001), while for problematic alcohol use a significant interaction term was found for gender and peer physical victimization (*b* = − 0.06; *p* = .008), indicating significant variations in the observed associations between girls and boys. The estimated interaction term for sexual victimization related to PTSD was *b* = 0.52 (*p* < .001) for girls and *b* = 0.02 (*p* = .880) for boys; for parental victimization related to PTSD, it was *b* = 1.26 (*p* < .001) for girls and *b* = 0.08 (*p* = .530) for boys; and for peer victimization related to problematic alcohol use, it was *b* = 0.04 (*p* < .001) for girls and *b* = 0.10 (*p* < .001) for boys.

## Discussion

In this study, we drew on self-report data from a large, population-based sample of Norwegian adolescents to investigate whether different forms of victimization were related to *specific* psychosocial indicators, such as sexual health, or whether more *general* associations would be observed across several forms of victimization. Victimization experiences were measured by means of detailed questions in three different domains—sexual victimization, physical victimization by parents, and physical victimization by peers. The results showed that sexual health was related to sexual victimization but not to other forms of victimization, suggesting specific mechanisms between sexual victimization and sexual health. However, all three forms of victimization were related to mental health and substance use problems, indicating more general mechanisms involved in the ways different forms of victimization may relate to health indicators in these domains.

The results show that knowledge about experiences with sexual victimization is relevant when assessing sexual health among adolescents. In our study, sexual victimization (defined as forced/coerced sexual encounters) was found to be associated with a broad array of indicators of sexual health, such as early intercourse debut, a high number of sex partners, engaging in sex without contraception while intoxicated, and participating in sexual acts for payment. Parental and peer physical victimization were either weakly or not at all associated with the included sexual health indicators.

Sexual victimization has longitudinally been linked to a greater total number of intercourse partners (B. J. Young et al., 2012), suggesting that such experiences may be related to more risky “sexual projects” (Hirsch & Khan, 2020). Testa et al. (2010), for instance, showed that college women in the USA who had experienced sexual victimization in adolescence engaged in higher levels of sexual risk-taking in college, which increased their vulnerability to future victimization. Similarly, another study among U.S. college women by Walsh et al. (2013) showed that low perceived sexual control stemming from childhood sexual abuse was associated with both permissive attitudes toward sexual encounters when drinking alcohol and having sex in the context of alcohol use. A potential aftermath of both behaviors is an increased risk of alcohol-related sexual victimization. These findings illustrate how the associations between sexual victimization and sexual health indicators may be bi-directional. Our findings may be interpreted within such a framework. However, we cannot rule out that the included sexual health indicators are linked to behaviors caused by unmeasured factors, such as genetic variation, which, again, may lead to an increased risk of sexual victimization (Harden, 2014).

Studies also suggest that sexual victimization may be associated with increased depression, anxiety, and post-traumatic stress (Hailes et al., 2019; Lindert et al., 2014). The associations in our study between sexual victimization and emotional distress are consistent with such a hypothesis. Adding to this, we observed that sexual victimization was also associated with increased rates of problematic alcohol use, as well as with the use of illegal substances. Parental and peer physical victimization were similarly associated with mental health and substance use, which is also consistent with previous findings (Gilbert et al., 2009; Moore et al., 2017). However, the association between parental physical victimization and post-traumatic stress was observed for girls only, while peer physical victimization was more strongly associated with problematic alcohol use among boys than girls. Thus, stress reactions seem to be a more common response to victimization among girls, while boys turn to substance use. The use of psychoactive substances may be a way of regulating emotions and coping after victimization (Bird et al., 2019). However, heavy drinking and the use of illegal drugs are simultaneously linked to contexts and situations where victimization often occurs (Tutenges et al., 2020) and where an increased risk of new victimization experiences may exist (Testa et al., 2010). This vicious circle may result in greater internalizing symptoms, such as hopelessness, sadness, and depression, and adolescents with such characteristics may become targets of new victimization experiences even more often (Foshee et al., 2004).

In previous publications addressing violent victimization, and particularly sexual victimization, it has been extensively discussed how to interpret the size of the associations between victimizations and health correlates. For example, Rind and Tromovitch (2007) argued that effects of childhood sexual abuse on adult sexual adjustment in the general population (measured by correlation coefficients) are typically minor at most. However, other researchers have argued that identified associations are of practical and clinical relevance (e.g., Najman et al., 2007a), highlighting how correlation coefficients are average measures of associations across all values of all variables, thus masking important effects at the extreme ends of the distribution. We acknowledge that the correlations in our study between victimization experiences and the included health indicators are small. However, the pattern of results identified in our study, with variation in associations between different forms of victimization and the health indicators, provides important information about the specificity of associations dependent on the type of victimization.

### Strengths and Limitations

The findings in this study are based on analyses of a large-scale, population-based sample of Norwegian 18- and 19 year-olds, enabling methodologically sound analyses of a relatively low-prevalent phenomenon, such as sexual victimization. Information on victimization was collected using behaviorally specific questions, which is the recommended approach in research on victimization (Krebs, 2014). The study further limited the assessment of sexual victimization to forced or coerced experiences, thus not confounding such victimization experiences with voluntary sexual relations that represent statutory violations. However, this study has several limitations. First, the data were cross-sectional; hence, we were unable to provide information about the directionality of the observed associations. Genetic influences may lead to an earlier initiation of sexual behavior, thereby increasing the risk of victimization experiences (Harden, 2014). We therefore acknowledge the potential of residual confounding due to genetic influences and other unmeasured factors influencing both victimization and the included health indicators. Second, the sample consisted of students who were in their final year of senior high school in Norway, that is, mostly those preparing for enrollment in higher education. Students who were in vocational practice or who had dropped out of school were not included in the survey. Thus, the diversity composition of the study sample in terms of social background is less than ideal. Third, the study did not contain information on emotional abuse, neglect, or verbal abuse, which in previous studies have been identified as important factors for the overall negative impact of victimization. Fourth, the instrument on intercourse debut age may be confounded by the respondents’ previous experiences with sexual victimization if they considered victimization experiences involving penetration as their intercourse debut. Finally, information on victimization was assessed retrospectively, which may have influenced the precision of the information. Nevertheless, Fergusson et al. (2011) found negligible bias in studies on the associations between retrospective reports of childhood maltreatment and adult life outcomes.

### Suggestions for Future Research

The present study points in the direction of several important topics for future investigations. In particular, the associations between sexual victimization and normative sexual behavior, early transitions into sexual relations, and sexual health should be further unpacked. Investigations on whether sexual victimization influences transitions into normative sexual behavior would also be welcome to garner a deeper understanding of the links between victimization and sexual health. Such investigations should also include digital sexual victimization and digital sexual practices. Longitudinal studies are especially relevant to this end, as they may yield information on both early predictors of and pathways to good and poor sexual health. Qualitative studies may additionally increase our understanding of how victims of violence interpret and handle their experiences. In this respect, not only should the timing of different sexual experiences be studied but also the reasons that adolescents do or do not engage in different sexual behaviors. Hirsch and Khan (2020) used the term “sexual projects” to highlight various reasons behind why young people have sex and highlighted how such projects are influenced by both local youth cultures and broader cultural currents.

## Conclusion

Our findings highlight the need to consider sexual victimization when assessing adolescent sexual health. Adolescent sexual health policies should have a broader scope than reproductive health and include services for young victims and initiatives to cultivate adolescents’ sexual citizenship more generally. Sexual citizenship, as defined by Hirsch and Khan (2020), is fundamental to sexual health, as it addresses “the capacity for sexual self-determination in all people, enabling them to feel secure, capable, and entitled to enact their sexual projects, and simultaneously insisting that all recognize others’ right to self-determination” (pp. xvi–xvii). How these capacities can be cultivated through sex education and youth health policies should, therefore, be high on the public health agenda.

### Supplementary Information

Below is the link to the electronic supplementary material.

Supplementary file1 (DOCX 20 kb)

## Data Availability

The data from the study and the full reproducible code of all the analyses are available at https://osf.io/d8fcq.
