# Supplementary material for: Sexual and Physical Victimization and Health Correlates Among Norwegian Adolescents
Source: Arch Sex Behav. 2023 May 8;52(7):2767–77. doi: 10.1007/s10508-023-02604-8 (PMC10684397; doi:10.1007/s10508-023-02604-8)
Supplement: Supplementary file 1 — Supplementary file1 (DOCX 20 kb) [file 10508_2023_2604_MOESM1_ESM.docx]

Supplementary Table 1

List of Items Used in Instruments on Victimization

| Instrument | Stem | Item |
| --- | --- | --- |
| Sexual victimization before/after the age of 13 | Have you been exposed to any of the following against your will? | Someone has pressured you to sexual acts against your will |
|  |  | Tried to force you to sexual intercourse or oral sex |
|  |  | Been threatened or forced to have sexual intercourse |
|  |  | Been threatened or forced to have oral sex |
|  |  | Been threatened or forced to have anal sex |
|  |  | Putted fingers or objects in your vagina or anus |
|  |  | Someone has had sex with you against your will when you were asleep or too drunk to resist |
| Parental physical victimization | Has your mother/father or stepmother/stepfather done any of the following towards you? | Pushed or shook you violently |
|  |  | Pulled your hair or pinched you |
|  |  | Slapped you |
|  |  | Hit you with the fist |
|  |  | Hit you with an object |
|  |  | Beaten you up |
|  |  | Done any other violent act towards you |
| Peer physical victimization | Has anyone at your own age exposed you to any of the following before/after the age of 13? | Getting hit without getting bruises |
|  |  | Getting bruises or injuries as a result of violence, without the need of medical assistance |
|  |  | Being injured so badly due to violence that you needed medical assistance |
